# The Substance Use Treatment and Recovery Team (START) study: protocol for a multi-site randomized controlled trial evaluating an intervention to improve initiation of medication and linkage to post-discharge care for hospitalized patients with opioid use disorder

**DOI:** 10.1186/s13722-022-00320-7

**Published:** 2022-07-28

**Authors:** Allison J. Ober, Cristina Murray-Krezan, Kimberly Page, Peter D. Friedmann, Karen Chan Osilla, Stephen Ryzewicz, Sergio Huerta, Mia W. Mazer, Isabel Leamon, Gabrielle Messineo, Katherine E. Watkins, Teryl Nuckols, Itai Danovitch

**Affiliations:** 1 RAND Corporation, 1776 Main St, Santa Monica, CA 90407-2138 USA; 2 University Pittsburgh, Pittsburgh, PA USA; 3 University of New Mexico Hospital, Albuquerque, NM USA; 4 University of Massachusetts Chan Medical School-Baystate, Springfield, MA USA; 5 Stanford University, Palo Alto, CA USA; 6 Cedars Sinai Medical Center, Los Angeles, CA USA

**Keywords:** Opioid use disorder (OUD), Medications for opioid use disorder (MOUD), Addiction consult team, Collaborative care, Linkage to follow-up, Inpatient

## Abstract

**Background:**

People with opioid use disorder experience high burden of disease from medical comorbidities and are increasingly hospitalized with medical complications. Medications for opioid use disorder are an effective, life-saving treatment, but patients with an opioid use disorder admitted to the hospital seldom initiate medication for their disorder while in the hospital, nor are they linked with outpatient treatment after discharge. The inpatient stay, when patients may be more receptive to improving their health and reducing substance use, offers an opportunity to discuss opioid use disorder and facilitate medication initiation and linkage to treatment after discharge. An addiction-focus consultative team that uses evidence-based tools and resources could address barriers, such as the need for the primary medical team to focus on the primary health problem and lack of time and expertise, that prevent primary medical teams from addressing substance use.

**Methods:**

This study is a pragmatic randomized controlled trial that will evaluate whether a consultative team, called the Substance Use Treatment and Recovery Team (START), increases initiation of any US Food and Drug Administration approved medication for opioid use disorder (buprenorphine, methadone, naltrexone) during the hospital stay and increases linkage to treatment after discharge compared to patients receiving usual care. The study is being conducted at three geographically distinct academic hospitals. Patients are randomly assigned within each hospital to receive the START intervention or usual care. Primary study outcomes are initiation of medication for opioid use disorder in the hospital and linkage to medication or other opioid use disorder treatment after discharge. Outcomes are assessed through participant interviews at baseline and 1 month after discharge and data from hospital and outpatient medical records.

**Discussion:**

The START intervention offers a compelling model to improve care for hospitalized patients with opioid use disorder. The study could also advance translational science by identifying an effective and generalizable approach to treating not only opioid use disorder, but also other substance use disorders and behavioral health conditions.

*Trial registration:* Clinicaltrials.gov: NCT05086796, Registered on 10/21/2021.

https://www.clinicaltrials.gov/ct2/results?recrs=ab&cond=&term=NCT05086796&cntry=&state=&city=&dist =

## Background

The US opioid epidemic continues to be of urgent national concern. Between 1999 and 2019, nearly 500,000 people died from an overdose involving opioids [1]. In 2020 and 2021, coincident with the COVID-19 pandemic, fatal and non-fatal opioid-related overdoses increased even more rapidly than in previous years [2–4]. People with opioid use disorder (OUD) experience high burden of disease from medical comorbidities [5] and are increasingly hospitalized with medical complications related to OUD [6–8]. Between 2002 and 2012, annual hospitalizations for OUD in the US nearly doubled, from 301,707 to 520,275, with inpatient charges for these hospitalizations nearly quadrupling [8]; by 2018, the number of inpatient stays related to OUD reached an estimated 748,900 [9]. Medications for opioid use disorder (MOUD) are highly effective and help reduce overdose rates, criminal behavior, infectious disease, and mortality [10–12] and are the standard of care for people with OUD, but patients with an underlying OUD admitted to the hospital seldom initiate MOUD while in the hospital or are linked with outpatient treatment for their OUD after discharge [13–15]. High rates of patient directed discharges among people with OUD (about 15%) suggest failure to address issues related to OUD such as opioid withdrawal and pain while in the hospital [16] and also lead to failed transitions to follow-up care after hospital discharge [17]. Between 2011 and 2015, about half a million hospitalization discharges per year included a diagnosis of OUD without provision of treatment or prevention services [18]. Failing to address OUD while patients are in the hospital either for a complication related to their OUD or for another illness or injury is a missed opportunity to initiate critical and life-saving treatment and leaves patients at high risk of continued use, delays in care, overdose, and costly readmission [6, 14, 17, 19–21].

Hospitalization is a critical time to identify patients with OUD and to initiate evidence-based treatments [16, 22]. Starting MOUD in the hospital and linking patients with post-discharge care addresses a common treatment gap and could improve patient outcomes and lower readmissions and costs. Some studies suggest that the inpatient hospitalization is a reachable moment when patients with OUD may be willing to engage with treatment, including initiating MOUD, if barriers can be reduced [23–33]. Although inpatient physicians frequently manage clinical conditions related to OUD, such as acute overdose, withdrawal, and infectious diseases, many report lacking knowledge and skills for addressing OUD [34, 35]. Given pressures to minimize length of stay in the hospital on the acute cause of admission, hospital teams may defer addressing chronic conditions like OUD. Moreover, few hospitals have established organizational infrastructure to support effective treatment of OUD, such as addiction focused consultative teams, evidence-based protocols, or the ability to coordinate care transitions needed to link patients to community resources [36]. Stringent federal privacy regulations, prescribing, dispensing and tracking regulations, insufficient training and reimbursement issues, create additional barriers [37–39]. Not least of all, patients with OUD often may not perceive the need to start treatment [40–42], and they may also experience stigma in health care settings [43, 44], leading to even greater ambivalence.

A hospital-based addiction consultation service has the potential to increase delivery of MOUD to patients with OUD (as well as other substance use disorders) during their hospitalization and link them to treatment after hospital discharge [45]. Prior studies suggest that an inpatient addiction consult team may have a positive effect on MOUD initiation and linkage to post-discharge care [34, 46] and result in lower readmission rates [47], and that this type of team is feasible, acceptable to patients and providers, and cost-effective to implement [34, 48–53]. Additionally, studies also show that patients who initiate MOUD in the hospital are more likely to continue MOUD for their OUD after discharge. [54] However, while these descriptive, observational, and quasi-experimental studies [29, 31, 47, 49, 53, 55–63] provide high-quality evidence, there have been no randomized controlled trials (RCTs) to test effectiveness of this model specifically for patients with OUD. RCTs can add definitive evidence to inform decisions on adoption of models of care, which is particularly valuable in a resource constrained health care system [64].

This article describes the protocol for a multi-site, RCT being conducted in three diverse hospitals in the United States to test whether an inpatient addiction consult team informed by the collaborative care model [65, 66] and evidence-based tools and resources, including motivational interviewing [67] and focused discharge planning [68, 69], improves MOUD initiation and linkage to post-discharge care for people with OUD compared to usual care.

### Study objectives and specific aims

This study will evaluate whether an addiction consult team called the Substance Use Treatment and Recovery Team (START) increases initiation of any US Food and Drug Administration (FDA)-approved MOUD (buprenorphine, methadone, naltrexone) during the inpatient stay, and increases linkage to treatment after discharge among hospitalized patients with OUD, compared to patients receiving usual care. Secondary outcomes include having an OUD-specific discharge plan, post-discharge MOUD and medical care utilization, and past 30-day opioid use. We hypothesize that compared to usual care, a higher proportion of patients in the START arm will initiate MOUD in the hospital; have linkage to post-discharge OUD treatment, including MOUD and psychotherapy; have an OUD-specific discharge plan; will receive any medical care; and will have fewer days of opioid use.

## Methods

### Study design and setting

**Fig. 1:**
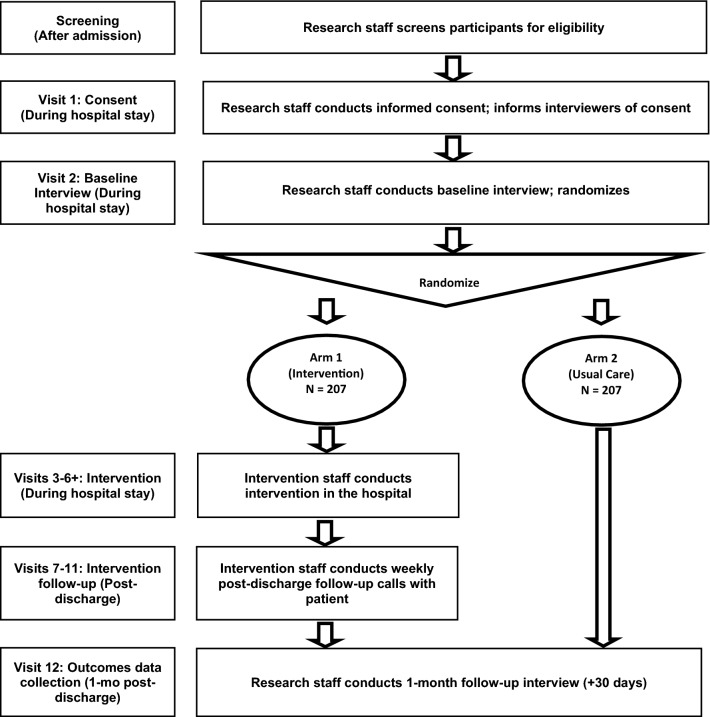
SPIRIT (Standard Protocol Items: Recommendations for Interventional Trials) flow diagram

This study is a three-site, pragmatic randomized controlled trial (RCT) testing the effects of START versus usual care (UC) on primary and secondary outcomes see Fig. 1 for study flow diagram. The trial is being conducted at Cedars-Sinai Medical Center (CSMC) in Los Angeles, the University of New Mexico (UNM) Hospital in Albuquerque, and Baystate Medical Center (BMC) in Springfield, Massachusetts. Patients are randomly assigned within each hospital to receive either START or UC, stratifying by any prior MOUD exposure and study site. Study outcomes are assessed through participant interviews at baseline and 1 month after discharge and data from hospital and outpatient medical records.

### Participants

The study will enroll 414 patients across all three hospitals over the course of approximately 10 months. (Our timeline may extend beyond 10 months due to delays associated with the COVID-19 pandemic). In order to be eligible for the study, individuals must be current inpatients at one of the three participating hospitals; be 18 or older; have a probable OUD diagnosis, defined by scores of  > 3 on the heroin or prescription opioid section of the World Health Organization Alcohol, Smoking, and Substance Involvement Screening Test (ASSIST) [70]; speak English or Spanish as a primary language; have a life expectancy of greater than 6 months (i.e., they are not in hospice); and be able to provide informed consent. Participants already receiving MOUD during their hospitalization will not be eligible for the study.

### Study conditions

#### Intervention condition: START

The START is an addiction consultation team comprised of an addiction medicine specialist (AMS) and care manager (CM) utilizing evidence-based interventions for OUD. The START provides diagnostic assessments, makes appropriate treatment recommendations, assists with implementation of treatment plans, establishes OUD-focused discharge plans, and facilitates linkage to treatment after discharge. The START is informed by the principles of the collaborative care model, a team-based treatment approach typically delivered by a physician-care manager team that has been found effective in health care settings for increasing use of evidence-based care and improving patient behavioral and substance use disorder outcomes but has not been previously adapted for a hospital-based addiction consult team [65, 66, 71, 72]. Collaborative care principles that inform this model include a patient-centered care team, population-based care that tracks patients using a registry, and use of evidence-and measurement-based care [73]. The START consists of an addiction medicine specialist (AMS) and a care manager (CM) who use a tailored intervention consisting of evidence-based tools and resources to overcome barriers to MOUD initiation and linkage to follow-up care. Table 1 details evidence-based tools and resources the START uses to address barriers to MOUD and linkage.

**Table 1:** How the START addresses treatment barriers for inpatients with OUD with evidence-based tools and resources

OUD care needed	Barrier	START component	Evidence-based tools and resources
Diagnosis and assessment for OUD, pain, withdrawal and psychosocial issues	Primary medical team focused on acute issues and may not identify or provide treatment for the underlying OUD	CM and AMS trained to assess OUD and relevant comorbidities, and to address key problems during the hospitalization in a non-judgmental and respectful way; whole person focus	DSM-5 diagnostic criteria [102]
			ASAM level of care criteria tool [103]
Motivational interviewing/harm reduction/trauma-informed care	Patient ambivalence, disempowerment and perceived stigma; mistrust	CM uses motivational interviewing, psychoeducation and trauma-informed care	Brief Negotiated Interview (BNI) [104, 105], adapted for hospital setting
			Education about safe injecting practices and overdose, provision of intranasal naloxone at discharge
			Culturally appropriate trauma-informed care [77]
Assessment of indications for MOUD	Inpatient provider lack of knowledge about MOUD, training and protocols	AMS with DEA X-waiver; MOUD protocols	ASAM national practice guideline for the use of medications in the treatment of addiction involving opioid use [103]
			Protocol for use of MOUD in the inpatient setting (adapted from California Bridge Project) [81]
OUD-focused discharge planning	Poorly coordinated care transitions; discharge planning not OUD-specific	CM uses adapted evidence-based discharge planning protocol and facilitates appropriate communication between key medical providers	Project Reengineered Discharge (RED), adapted for patients with OUD [69]
			Electronic registry to monitor protocol delivery and track patients after discharge [73]
Access to post-discharge OUD care	Limited outpatient capacity	Rapid-access discharge pathways set up	Relationships with community OUD providers to establish rapid-access discharge pathways, resource lists

The START CM and AMS have interrelated roles providing patient care, at times providing recommendations to the primary medical team, at other times directly delivering services; responding to specific challenges related to addiction and its bio-psycho-social implications; and overseeing clinical team-based care regarding the patient’s OUD. Each AMS is a physician who holds a DEA X-waiver and/or board certification in addiction medicine or psychiatry. The AMS conducts a medical assessment, including withdrawal potential, relapse risk, and relevant comorbidities that influence medical management of OUD, and evaluates whether the patient is a candidate for MOUD. FDA-approved MOUDs include methadone, buprenorphine/naloxone, and naltrexone. If appropriate for MOUD, the AMS discusses the treatment with the patient and the patient’s medical team and either will provide consultative guidance (if requested by the medical team) or prescribe the medication. The AMS provides ongoing clinical supervision to the CM and is available to communicate with aftercare providers to support continuous MOUD. The CM and AMS discuss patient care in terms of diagnosis, motivation for change, treatment and aftercare planning, barriers, and potential solutions. For patients in the one-month follow-up period, the CM provides updates to the AMS on measurement-based care elements including withdrawal symptoms, substance use, MOUD adherence, and side effects.

The CM (START CMs have an MSW, LCSW, and/or more than 5 years of experience working with people with substance use disorders) delivers an adapted Brief Negotiated Interview [(BNI); a structured, evidence-based approach designed to improve readiness for substance use disorder treatment based on motivational interviewing (MI)] [74–76] to engage, assess, and help motivate the patient to initiate treatment and/or post-discharge care for their OUD; provides educational information to the patient about MOUD, psychosocial interventions, and overdose prevention; conducts psychosocial assessments and assesses for risk factors; and guides the patient through safety planning and crisis management as needed. Working with the AMS and the primary medical team the CM also works with the patient to develop an OUD-focused discharge plan using techniques and materials adapted from Project Reengineered Discharge (RED), an evidence-based discharge planning protocol [68], that include active planning and teach-back techniques, facilitated linkage to follow-up care, and post-discharge follow-up. For patients who do not initiate MOUD and do not wish to obtain follow-up care, the CM addresses harm reduction needs and helps facilitate linkage if the patient’s readiness changes. The CM uses a registry to track treatment and follow-up and to prioritize care based on the patient’s level of need.

The START “starts” where the patient is; that is, the START respects patients’ thoughts and feelings about their opioid use, does not confront them about their use, and does not try to persuade them to initiate MOUD or other treatment. Consistent with the BNI, the AMS and CM use a MI style in their approach to talking with patients [76]. MI is a client-centered, directive but non-confrontational counseling style for eliciting behavior change. The examination of ambivalence around behavior change is a central tenet of MI. The AMS and CM use MI in their encounters with the patient to help them resolve ambivalence about starting treatment for an opioid use disorder. The START also recognizes that personal and cultural backgrounds inform patients’ experiences with opioid use and treatment. The START practices trauma-informed care and cultural humility. Trauma-informed care involves engaging in shared decision-making, building trust, empowering patients, and creating a safe environment to respond to trauma in ways that are culturally and linguistically appropriate [77]. Nearly half of people with OUD have a lifetime history of post-traumatic stress disorder [78, 79], which makes addressing trauma an especially important part of care for this population. Cultural humility is a part of trauma-informed care, and it is crucial for providing equitable, effective care to diverse populations [80].

**Fig. 2:**
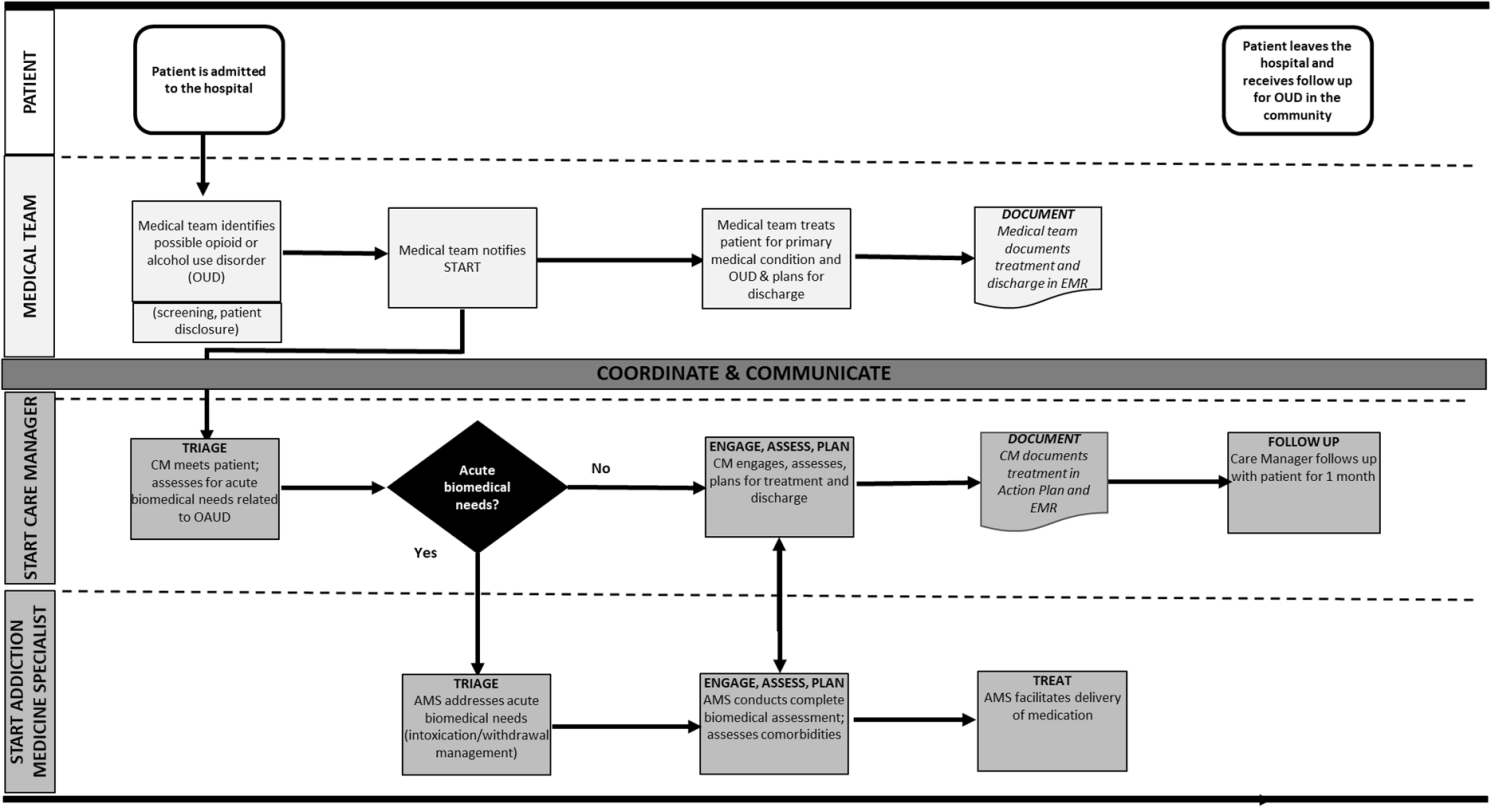
START Workflow

The components of the START workflow are as follows (see Fig. 2):

1. **Triage** The CM or AMS assesses the patient’s acute biopsychosocial stability and prioritizes interventions in accordance with clinical status and hospital course. Some patients may need an urgent intervention to address active withdrawal, or counsel to prevent a patient directed discharge. For other patients, intervention is deferred while acute medical conditions are stabilized.
2. **Engage, assess, plan** If there is not an urgent need for medical intervention or after the urgent medical need is addressed, the CM and/or AMS:
  Engages with the patient (CM and AMS)
  Conducts a diagnostic and biopsychosocial assessment (CM)
  Conducts a biomedical assessment and addresses comorbidities (AMS)
  Delivers the adapted BNI to assess and increase readiness for treatment and develops a plan for initiating evidence-based treatment (MOUD, psychotherapy) during and after the hospital stay (CM)
  Ensures the patient understands the follow-up plan and addresses barriers (CM)
3. **Treat** Treatment includes:
  Facilitating appropriate management of intoxication, withdrawal symptoms, comorbidities, and MOUD (AMS)
  Facilitating psychosocial treatment for OUD, if indicated and available (CM)
  Educating patients about harm reduction strategies (CM), including use of overdose reversal kits (CM/AMS)
4. **Communicate and Coordinate **
  The CM and AMS communicate with each other to continue care throughout 1 month after the patient is discharged
  The CM and AMS communicate with the patient and medical team, and, when appropriate, the patient’s family and outpatient providers
5. **Follow up** The CM calls the patient once a week for 1 month after the patient is discharged from the hospital to assess whether the patient is following through with the discharge plan. The CM may also call outpatient providers to facilitate linkage to care.

#### UC study condition

UC consists of each hospital’s current practices for managing patients identified with OUD along with each patient enrolled in the study receiving MOUD education and referral information. We use UC as the comparator because there are no other evidence-based interventions for achieving our proposed outcomes. None of the hospitals currently employs an addiction consult team that consists of an AMS and CM that systematically uses a set of principles based on collaborative care along with evidence-based tools and resources (e.g., motivational interviewing, adapted BNI and Project RED resources) to support patients with OUD. A CM and AMS at each hospital serve as CM and AMS for the START study and will not see UC patients during the study period. At CSMC, patients randomized to the UC study condition may receive a referral to the existing consultation liaison (CL) psychiatry service if the patient’s medical team determines the need for a consult, or they will be treated and provided discharge planning directly by the medical team. The CSMC CL service has clinicians who can discuss opioid use with the patient and help the patient initiate medication, if indicated. These usual CL service providers also can provide consultation to the medical team on whether medication initiation in the hospital and treatment after discharge are indicated. At UNM and BMC hospitals, patients randomized to the UC study condition can be treated directly with MOUD and provided discharge planning by the medical team. At BMC Hospital, the referring physician will have the option of contacting the standard psychiatric CL or addiction consult service for patients in the UC study condition, which will not include an AMS or CM. If the START AMS or CM at any hospital is approached by a member of the medical team for consultation on an OUD patient, they will refer them to the California Bridge Program Tools and Resources website [81].

### Study procedures

#### Inclusion and exclusion criteria

Inclusion criteria are as follows: (1) admitted to an inpatient bed at CSMC, UNM Hospital, or BMC; (2) age 18 and older; (3) have a probable OUD diagnosis, defined by scores of  > 3 on the opioid section of the Alcohol, Smoking, and Substance Involvement Screening test (ASSIST) [70]; (4) speaks English or Spanish as primary language; (5) able to provide informed consent. An individual who meets any of the following criteria is excluded from participation in this study: (1) already receiving FDA-approved medication treatment for an opioid use disorder in the hospital, defined as not being on MOUD at the time the patient is approached by the study team; (2)  < 6 months life expectancy.

#### Patient identification and recruitment

Approved study staff prescreen patients for screening and potential enrollment through a daily electronic medical record (EMR) report of risk factors for opioid use disorder that lists potentially eligible subjects (variables include demographics, opioid history, diagnoses, and screenings) and through clinician referral to the study. Upon consent from the requesting medical team (required at two of the three hospitals), study staff conduct eligibility screening. Screening is conducted in person or remotely using an approved and secure web-based data capture system (REDCap) [82] housed at the study statistics and data coordinating center (SDCC) at UNM.

#### Consent, baseline and follow-up interviews, randomization

Study staff conduct the informed consent process including reviewing the full consent form and/or the consent summary with the patient. Consent is obtained via electronic signature. All patients are given patient education materials on OUD and harm reduction. Approved study staff conduct an in-person or remote 30–40 min baseline interview. Interview data are recorded on a tablet or computer into REDCap. Each site is responsible for remunerating their participants $50 per their institutional practice. Following the baseline interview, approved study staff randomize the patient to the START or UC arm by accessing a site-specific randomization module in REDCap. Study staff randomize participants into START or UC using stratified, block randomization, stratified by site and prior MOUD exposure, and using randomly permuted block sizes of 2, 4, and 8 (all programmed into REDCap). All patients enrolled in the study receive information on OUD and MOUD, and on how and where to receive services. Enrollment is continuous with the goal of reaching the desired sample size (N = 414); some sites may enroll more or less than others. Interviewers from the RAND Corporation Survey Research Group (SRG) conduct a follow-up interview by telephone 1 month after the patient is discharged from the hospital, within a 2 month follow-up window. The UNM SDCC provides contact information to RAND SRG through secure REDCap access. The follow-up interview is 30–40 min, and RAND SRG remunerates participants $50 per each hospital’s practices. See Table 2 for SPIRIT (Standard Protocol Items: Recommendations for Interventional Trials) schedule of enrollment, interventions, and assessments.

**Table 2:** SPIRIT (Standard Protocol Items: Recommendations for Interventional Trials) schedule of enrollment, interventions, and assessments

Timepoint	Study period				
	Enrollment	Post-enrollment			
	−T1	T1	0		T2
	Screening, consent	Baseline	Intervention (during hospital stay)	Intervention follow-up (post-discharge)	Follow-up interview (30–60 days post-discharge)
Eligibility screen	X				
Informed consent	X				
Randomization		X			
Interventions					
Intervention 1	X	X	X	X	X
Control	X	X			X
Assessments					
ASSIST	X				
Demographics	X				
MOUD utilization	X				X
Employment		X			X
Depression (PHQ-9)		X			X
Anxiety (GAD-7)		X			X
Social support (MSPSS)		X			
Overdoses		X			X
Pain intensity and frequency (PEG)		X			X
30-day opioid (and other substance) use (adapted from NSDUH)		X			X
SUD treatment utilization (adapted from NSDUH)		X			X
SUD healthcare and mental health utilization (adapted from GAIN)		X			X
Opinions about MOUD		X			
Severity of substance use (PROMIS)		X			X
Patient experience of stigma		X			
Significant other with OUD		X			
Criminal justice involvement		X			
Patient experience of chronic illness care (PACIC)					X
Therapeutic alliance (CAHPS)^a^					X
Satisfaction with START^a^					X

^a^Intervention group only

### Measures

#### Outcome variables

We provide our primary and secondary outcome variables and endpoints in Table 3.

**Table 3:** Outcome variables and endpoints

Outcome	Endpoint
Primary	
In-hospital initiation of MOUD	Proportion of patients in each arm who initiate MOUD prior to discharge, defined as use of any FDA-approved pharmacotherapy for OUD, including buprenorphine, naltrexone and methadone
Linkage to follow-up OUD care	Proportion of patients in each arm who attend at least one OUD-related care visit within 30 days of hospital discharge
Secondary	
OUD-specific discharge plan	Proportion of patients in each arm with an after-hospital care plan that specifies a date and time for a post-discharge addiction care appointment
Any post-discharge MOUD utilization	Proportion of patients in each arm who initiate MOUD or continue MOUD treatment within 30 days following hospital discharge
Post-discharge outpatient medical care	Proportion of patients in each arm who complete at least one visit to an outpatient medical provider within 30 days of hospital discharge
Past 30-day number of days with any opioid use	Mean (or median, depending on distribution) days of use in the past 30 days after hospital discharge

#### Measures

We included measures of demographics, mental health symptoms, social support, medical symptoms and treatment, substance use treatment history, opinions about MOUD, experience of chronic illness care, and opinions about the START intervention. Table 4 shows all measures and data sources for outcomes and potential covariates, mediators, and moderators.

**Table 4:** Measures

Variable	Measure	Data source
Sociodemographics		
Age, sex (assigned at birth), gender identity, hispanic ethnicity, race, housing status	N/A	Eligibility screener
Marital status, income, education and insurance type	N/A	Baseline interview
Mental health status and symptoms		
Prior psychiatric diagnosis (bipolar disorder or schizophrenia)	N/A	Eligibility screener
Prior psychiatric hospitalization		
Depression (9 items)	PHQ-9 [106, 107]; GAD-7 [108–110]	Baseline interview 1-month follow-up
Anxiety (7 items)		
Social support		
Social support: family, friends, significant other (6 items; 2 each scale)	Modified multidimensional scale of perceived social support [111]	Baseline interview 1-month follow-up
Medical symptoms/treatment		
Overdoses (lifetime, past 3 mos)	N/A	Baseline interview 1-month follow-up; EMR
Primary and secondary diagnosis (inpatient stay)	Medical or mental health conditions as determined by the inpatient physician	EMR
Pain intensity and duration	PEG [112]	Baseline interview 1-month follow-up
Length of hospital stay	Days in hospital	EMR
Substance use treatment history		
Ever used MOUD; times started an MOUD; type of MOUD; other treatment	N/A	Eligibility screener
Recent substance use treatment utilization; opinions; consequences; stigma		
SUD treatment utilization	Adapted from National Survey on Drug Use and Health (NSDUH) [113]	Baseline interview 1-month follow-up (validation through follow-up with service provider)
Healthcare utilization (ER, inpatient, outpatient) related to SUD (5 items)	Adapted from Global Appraisal of Individual Needs (GAIN) [114]	Baseline interview 1-month follow-up
Opinions about MOUD	Adapted opinions about MAT (OAMAT) [115]	Baseline interview
Severity of substance use	PROMIS	Baseline interview 1-month follow-up
Patient experience of stigma	Adapted from Grosso et al. [116]	Baseline interview
Patient experience of chronic illness care (11 items)	Patient Assessment of Chronic Illness Care (PACIC) [117]	1-month Follow-up
Criminal justice involvement	Locally developed	Baseline interview
Intervention—related		
Intervention “dose”; exposure	Amount time spent with patient number of encounters with patient	START registry (Deidentified)
Therapeutic alliance	Consumer Assessment of Healthcare Providers and Systems (CAHPS^®^) [118]	1-month follow-up (START only)
Satisfaction with START intervention	Locally developed	1-month follow-up (START only)

#### Intervention fidelity measures

Fidelity to the START intervention key components (collaborative care, the brief negotiated interview, and addiction-focused discharged planning) as well as competency in using MI will be measured. Table 5 shows our fidelity and MI competency measures***.***

**Table 5:** Fidelity and competency measures

Domain	Measure
Collaborative care processes [119]	
CM visit	Proportion of patients who saw CM at least one time
AMS visit	Proportion of patients who saw AMS at least one time
CM/AMS consultation	Proportion of patients who were discussed at least one time by CM and AMS
Follow-up	Proportion of patients who got at least 1 follow-up within 4 weeks after discharge
Evidence-based care: BNI [67]	
	Proportion of patients who got pros and cons
	Proportion of patients who got the readiness ruler
	Proportion of patients who got an OUD-focused action plan
Evidence-based care: RED [68, 69]	
	Proportion of patients for whom CM reviewed action plan prior to discharge
	Proportion of patients who reported that CM reviewed action plan in a way that they understood
	Proportion of patients for whom a follow-up appt was made prior to discharge
Competency using MI	
Baseline	CM and AMS reached “good” MI competency at baseline Motivational Interviewing Treatment Integrity scale (MITI) [120] (recorded interviews)
Midpoint	CM and AMS reached “good” MI competency at midpoint (recorded interviews)

### Data safety and monitoring board (DSMB)

The University of California Los Angeles (UCLA) Data and Safety Monitoring Board for Addiction Medicine (DSMBAM) serves as the DSMB for this stuy. DSMBAM members are multidisciplinary and include expertise in addiction medicine, biostatistics, basic science, epidemiology, clinical trail methodology, and biomedical ethics.

### Data management and quality control

Data will be collected from multiple sources throughout the course of the study. All prospectively collected data will be directly entered into the UNM REDCap electronic data capture system which is administered by the UNM Clinical and Translational Science Center (CTSC). The UNM Statistics and Data Coordinating Center (SDCC) will develop electronic data collection forms of the patient interviews in REDCap. All data will be stored on UNM’s secured servers and behind their firewall. Other data sent to UNM will be transferred via SFTP following all institutional policies and executed data use agreements. The SDCC team at UNM will be responsible for data quality control, including evaluating data for adherence with the protocol and for accuracy. Site queries will occur every 2–4 weeks. Study status reports generated from the database will provide a basis for ongoing monitoring of subject accrual and retention, as well as completeness of data.

### Statistical analysis

Baseline characteristics will be summarized with descriptive statistics including means and standard deviations or medians and interquartile ranges for continuous variables, and frequencies and percentages for categorical variables. Summaries will be presented overall, by intervention arm, and stratified by previous MOUD exposure. Continuous baseline demographics and characteristics will be compared with *t* tests or Wilcoxon rank sum tests, as appropriate. Categorical variables will be compared with chi-square or Fisher exact tests, as appropriate. Corresponding confidence intervals will be reported in addition to p-values. The primary and secondary analyses will be performed for the intention-to-treat population, which consists of all randomized subjects. Every effort will be made to obtain all necessary outcome and covariate data. We will use inverse probability weighting and multiple imputation to adjust for missing covariate data [83]. Specifically, we will examine whether observable baseline characteristics differ by attrition status, and if so, we will adjust our comparisons using weights. Multiple imputation will be used to impute intermittently missing data for study completers. We will not impute data for outcomes, only for covariates.

#### Primary and secondary endpoint analysis

Unadjusted point estimates and confidence intervals for proportions and means will be reported by arm and by prior MOUD use for endpoints. Primary endpoints will be compared between arms by fitting a multivariable logistic regression model to each that includes as independent variables: intervention arm, prior MOUD exposure and site, as well as relevant baseline characteristics as covariates, including age, insurance status (as a marker for income), race, and ethnicity. Additional covariates that may be included are substance use severity, homelessness, length of index hospitalization, comorbid medical and psychiatric conditions, as well as any other variables also thought to be associated with outcomes that demonstrated imbalance between treatment arms [84]. Site will be included as a fixed effect to reflect the study design and to control for potential variability in START implementation. Odds ratios and their Bonferroni-adjusted 97.5% Wald confidence intervals will be reported for the two primary endpoints. Should the prevalence of outcomes be relatively high in both arms, log-binomial or Poisson regression models will be considered with risk ratios and their 97.5% confidence intervals reported, instead [85]. Similar analyses as described for the primary endpoints will be performed for these secondary proportions outcomes, but instead reporting 95% confidence intervals. A general linearized model to number of days of opioid use will be fitted along with the covariates described for the logistic regression models. An appropriate link function will be identified and used based on the distribution of the outcome data**.**

#### Exploratory analysis

Mixed findings in past research of consult services suggest that sex possibly could moderate START effectiveness [86–88]. We will conduct exploratory analyses to see if patient sex or gender, as well as race/ethnicity, has an effect on primary outcomes or retention. Adjusted odds ratios and their 95% confidence intervals will be calculated from interaction effects between treatment group and sex or gender from the specified linear models for the primary and secondary outcome measures. To explore possible mechanisms of how START works, we will conduct the following exploratory analyses: (1) assess the mediating effect of inpatient MOUD initiation on use of MOUD and linkage with OUD treatment post-discharge; (2) assess the mediating effect of completion of an OUD-specific discharge plan on linkage with OUD treatment 30 days post-discharge; (3) assess the moderating effects of patient characteristics (e.g., gender, race, ethnicity, insurance status, comorbid conditions, prior MOUD use) on medication initiation and post-discharge linkage. We will summarize bivariate relationships between site and patient characteristics. To evaluate how these relationships may affect endpoints, we will assess the interaction effects between site and these covariates from the regression models described for the primary and secondary analyses. Additionally, of interest is time to linkage to care following discharge. A Cox proportional hazards model will be fitted to the time to linkage with intervention arm and other relevant baseline characteristics as covariates, including age, insurance status (as a marker for income), race, and ethnicity. Additional covariates identified for the primary and secondary analyses may also be included. The proportional hazards assumption will be assessed. The relative risk and 95% CI for the two arms will reported and median times to linkage will be reported.

#### Sample size and power

A sample size of n = 414 (allowing for 20% attrition) and adjusted type I error rate of 2.5% provides 84% power to detect an odds ratio of 2.3 comparing the inpatient MOUD initiation rates in the START and UC arms, stratified on prior MOUD use. Based on literature, 14% of UC patients who are MOUD-naïve initiate MOUD in hospital [19]. Assuming the average of MOUD-naïve and MOUD-experienced inpatient MOUD initiation rates is 20%, we have an adequate sample size and power to detect this increase of inpatient MOUD initiation in the START arm (37%) compared to UC [19, 54, 89]. We base the sample size estimate on the linkage to care measure (Primary Endpoint 2) since the probabilities of successful linkage are lower than for inpatient MOUD initiation. Linkage to care rates reported in the literature range between 10 and 17% in usual care settings. To err on the side of caution, we estimate linkage to care in UC for MOUD-naïve and MOUD-experienced to be 5% and 10% [19, 54, 89, 90], respectively, yielding an average of 7.5%. We hypothesize that at least 20% of patients randomized to the START arm will link to OUD care (attend at least one OUD-related visit) within 30 days following discharge. Assuming a Bonferroni-corrected, two-sided type I error rate of 2.5% to adjust for two primary endpoints, we will enroll a minimum of 414 patients (207 in each intervention arm) to have 80% power to detect this difference. This estimate includes an adjustment for up to 20% attrition. This effect size corresponds to a clinically meaningful odds ratio of 3.0. Prior studies in different settings have found larger effects [54, 84, 90], supporting our ability to conduct this test. Sample size calculations for the primary endpoints were performed in PASS 14 using stratified Mantel–Haenszel tests for two proportions between two groups [91], with strata defined as 50% MOUD-naïve and 50% MOUD-experienced [54, 84, 90, 92, 93]. Due to the short 1 month duration of participation, subject withdrawal from the study is not anticipated to be significant.

## Discussion

The START, a collaborative care-informed consultative team, is proposed to increase adoption of evidence-based care and improve outcomes for hospitalized patients with OUD. Hospitals have extensive experience using care managers to improve in-hospital and follow-up care for several patient populations at high risk of readmission [94, 95], including acute medical patients [96], and some have a consultation service to support the medical team with patients in need of behavioral health care. More recently, addiction-focused consult teams have begun to emerge in hospitals across the United States and elsewhere [29, 31, 47, 49, 55–63]. However, patient-level randomized controlled trials are necessary to evaluate how addiction consult teams affect outcomes for hospitalized patients with OUD. The addiction consult team, along with the evidenced-based tools and resources adapted for the START intervention, offers a novel, comprehensive approach for facilitating MOUD initiation in the hospital and linking patients to follow-up care for OUD. While other consult services described in the literature have additional professionals such as peer navigators and nurses on the team, [97, 98] we chose to test a foundational low-resource model, as many hospitals do not have the volume of patients with OUD to justify larger, more complex multidisciplinary consultation services*.* In future research additional models can be tested to identify core team members and components.

Our study is a multi-site, randomized pragmatic trial that will enroll patients at three diverse academic hospitals, allowing for a real-world implementation context, which will inform and potentially accelerate translation of the START into practice. Moreover, the START has potential for high impact because it can both improve public health and advance translational science. The undertreatment of OUD is an important public health and translational science problem. In 2015, 11.5 million individuals reported misusing opioids, and 1.9 million reported being addicted to opioids [99], yet fewer than 20% received any treatment [101]. By experimentally testing the effects of the START, this study could both improve public health by identifying an efficient and generalizable model to increase OUD treatment delivery and decrease the downstream effects of untreated OUD. This study can also advance translational science by identifying an effective and generalizable approach to address translational roadblocks that result in the undertreatment of substance use disorders and behavioral health conditions in hospital settings.

## Data Availability

The datasets generated and analyzed during this study are not publicly available due to the sensitive nature of the data. They can be made available from the corresponding author on reasonable request and with execution of appropriate Data Use Agreements.

## Abbreviations

AMS: Addiction medicine specialist
ASAM: American Society of Addiction Medicine
ASSIST: Alcohol, smoking, and substance involvement screening test
BMC: Baystate Medical Center
BNI: Brief Negotiated Interview
CAHPS: Consumer Assessment of Healthcare Providers and Systems
CL: Consultation liaison
CM: Care manager
CSMC: Cedars-Sinai Medical Center
DEA: Drug Enforcement Administration
FDA: Food and Drug Administration
EMR: Electronic medical record
GAD-7: General anxiety disorder-7
GAIN: Global Appraisal of Individual Needs
MAT: Medication assisted treatment
MI: Motivational interviewing
MITI: Motivational interviewing treatment integrity
MOUD: Medications for opioid use disorder
MSPSS: Multidimensional scale of perceived social support
NSDUH: National Survey on Drug Use and Health
OAMAT: Opinions about MAT
OUD: Opioid use disorder
PACIC: Patient experience of chronic illness care
PEG: Pain, enjoyment, general activity
PHQ-9: Patient health questionnaire-9
RCT: Randomized controlled trial
SDCC: Statistics and data coordinating center
SPIRIT: Standard Protocol Items: Recommendations for Interventional Trials
SRG: Survey research group
START: Substance Use Treatment and Recovery Team
SUD: Substance use disorder
UC: Usual care
UNM: University of New Mexico
